# Effectiveness of a peer-led adolescent mental health intervention on HIV virological suppression and mental health in Zimbabwe: protocol of a cluster-randomised trial

**DOI:** 10.1017/gmh.2020.14

**Published:** 2020-08-28

**Authors:** Silindweyinkosi Chinoda, Abigail Mutsinze, Victoria Simms, Rhulani Beji-Chauke, Ruth Verhey, Joanna Robinson, Taryn Barker, Owen Mugurungi, Tsitsi Apollo, Epiphany Munetsi, Dorcas Sithole, Helen A. Weiss, Dixon Chibanda, Nicola Willis

**Affiliations:** 1 Friendship Bench, Harare, Zimbabwe; 2 Africaid, Harare, Zimbabwe; 3 MRC Tropical Epidemiology Group, London School of Hygiene and Tropical Medicine, London, UK; 4 Childrens’ Investment Fund Foundation, London, UK; 5 AIDS & TB Unit, Ministry of Health and Child Care, Zimbabwe; 6 Ministry of Health and Child Care, Zimbabwe and Mental Health Services, Zimbabwe; 7 Department of Psychiatry, University of Zimbabwe College of Health Sciences, Zimbabwe; 8 Centre for Global Mental Health, London School of Hygiene and Tropical Medicine, London, UK

**Keywords:** Adolescents, HIV, mental health virological suppression, peer-led

## Abstract

**Background:**

Adolescents living with HIV (ALHIV) experience a high burden of mental health disorder which is a barrier to antiretroviral therapy adherence. In Zimbabwe, trained, mentored peer supporters living with HIV (Community Adolescent Treatment Supporters – CATS) have been found to improve adherence, viral suppression and psychosocial well-being among ALHIV. The Friendship Bench is the largest integrated mental health programme in Africa. We hypothesise that combining the CATS programme and Friendship Bench will improve mental health and virological suppression among ALHIV compared with the CATS programme alone.

**Methods:**

We will conduct a cluster-randomised controlled trial in 60 clinics randomised 1:1 in five provinces. ALHIV attending the control arm clinics will receive standard CATS support and clinic support following the Ministry of Health guidelines. Those attending the intervention arm clinics will receive Friendship Bench problem-solving therapy, delivered by trained CATS. Participants with the signs of psychological distress will be referred to the clinic for further assessment and management. The primary outcome is HIV virological failure (≥1000 copies/ml) or death at 48 weeks. Secondary outcomes include the proportion of adolescents with common mental disorder symptoms (defined as Shona Symptom Questionnaire (SSQ-14) score ≥8), proportion with depression symptoms (defined as Patient Health Questionnaire (PHQ-9) score ≥11), symptom severity (mean SSQ-14 and PHQ-9 scores) and EQ-5D score for health-related quality of life.

**Conclusions:**

This trial evaluates the effectiveness of peer-delivery of mental health care on mental health and HIV viral load among ALHIV. If effective this intervention has the potential to be scaled-up to improve these outcomes.

Trial registration: PACTR201810756862405. 08 October 2018.

## Background

Whilst 80% of the estimated 64000 adolescents living with HIV (ALHIV) in Zimbabwe have been initiated on antiretroviral therapy (ART), a recent study of 500 adolescents on ART found 48% had virological failure (VF) (Mavhu *et al*., 2017). Globally, adolescents are the age group with the highest rate of HIV treatment failure, and the only age group in which HIV-related mortality is not decreasing (Joint United Nations Programme on HIV/AIDS (UNAIDS), 2016). This is partly due to the multiple challenges to adherence faced by ALHIV (Hudelson and Cluver, 2015) including late disclosure, poor treatment literacy, lack of support, stigma, pill fatigue and side effects.

Common mental disorders (CMDs, i.e. depression and anxiety) can inhibit adherence to medication including ART, possibly by their effects on self-efficacy and motivation(Wagner *et al*., 2017), as supported by Social Cognitive Theory and the Information, Motivation and Behavioural skills (IMB) model of health behaviour. However, the impact and management of depression among ALHIV, including its impact on ART adherence and viral suppression, have not been widely studied (Willis *et al*., 2018). Studies among ALHIV in sub-Saharan Africa have found an association between mental health disorders and poor ART adherence or VF (Lowenthal *et al*., 2012; Mutumba *et al*., 2016). In Zimbabwe, 63% of 141 ALHIV aged 13–18 were above the ‘at-risk threshold’ for CMD symptoms (Shona Symptom Questionnaire (SSQ-14) score ≥8), and those with CMD were less likely to have good reported ART adherence (*p* = 0.04) (Mavhu *et al*., 2013). In Johannesburg, 343 ALHIV aged 13–19 years accessing five paediatric ART clinics were assessed using standardised measures and found that 27% were symptomatic for depression, anxiety and post-traumatic stress disorder, and a further 24% indicated the signs of suicidality (Woollett *et al*., 2017).

Among Zimbabwean ALHIV, CMD symptoms are associated with poor self-reported ART adherence (Mavhu *et al*., 2013), and a pilot study found evidence of improved virological suppression following combined depression and adherence counselling (Abas *et al*., 2018). There is some evidence that treating CMDs can improve ART adherence and virological suppression in adults. Depression treatment was associated with improved ART adherence in a meta-analysis of 29 studies from the USA, although there was no evidence of an effect when only the 15 randomised controlled trials were included (Sin and DiMatteo, 2014). Evidence from Africa is scant. In Uganda, depression alleviation at 12 months was associated with improved ART adherence, and the relationship was mediated by adherence self-efficacy (Wagner *et al*., 2017). This result suggests that depression treatment may improve participants’ ability and confidence to take ART, which in turn improves adherence. ART adherence and virological suppression improved after depression treatment of 41 PLHIV in Cameroon (Gaynes *et al*., 2015).

In Zimbabwe, as in many other countries, there is limited understanding of the mental health needs of adolescents, including those living with HIV. There is also no system for the identification, referral and management of ALHIV and mental illness (Mangezi and Chibanda, 2010). With only 12 practising psychiatrists in Zimbabwe, innovative approaches to the provision of mental health services are essential. In the proposed trial, we will evaluate the impact of two existing psychosocial interventions delivered by lay workers on ART adherence among ALHIV.

The Zvandiri programme, developed by the Private Voluntary Organisation Africaid, is a model of differentiated clinical service delivery for children, adolescents and young people living with HIV in Zimbabwe and has been cited as a ‘best practice’ intervention by the WHO (Willis *et al*., 2018; World Health Organisation, 2019) and adopted or adapted in eight countries in the region. At the forefront of Zvandiri are trained, mentored peer supporters aged 18–24 years old and living with HIV. These peer supporters are known as Community Adolescent Treatment Supporters (CATS). CATS are integrated within the clinics and surrounding communities and generate demand for HIV services across the cascade (Willis *et al*., 2018), supporting ART initiation, adherence, linkage and retention in care for their caseload of children, adolescents and young people. This CATS model results in improved adherence, retention (Willis *et al*., 2019) and virological suppression (Mavhu *et al*., 2020) among ALHIV compared with adolescents receiving standard of care alone. CATS are actively engaged in the development and implementation of their own peer-led mental health intervention in response to the needs of their peers. To achieve this, they aim to build on their existing experience as peer counsellors.

The Friendship Bench is a low-intensity mental health intervention delivered by trained and supervised lay health workers (Chibanda *et al*., 2015). It is based on cognitive behavioural therapy principles with an emphasis on problem-solving therapy (PST) (Chibanda *et al*., 2011). A cluster-randomised controlled trial showed it was effective for treating symptoms of CMDs (i.e. depression and anxiety disorders), and improving quality of life, among adults (Chibanda *et al*., 2016d). Due to its success in the last 10 years, the Friendship Bench has been scaled-up to over 70 primary health care facilities in Zimbabwe (Chibanda *et al*., 2016c). The Friendship Bench is the only evidence-based mental health intervention currently scaled up in Zimbabwe (Chibanda *et al*., 2016c) and is being adopted by other countries including Malawi, Tanzania and the USA. Zvandiri aims to adopt it for its CATS by training and mentoring them in PST.

## Method

### Overall aims and study hypothesis

The aim of the trial is to evaluate the feasibility and effectiveness of a peer-led mental health support intervention on virological suppression, mental health and quality of life in ALHIV in Zimbabwe. We will evaluate whether training and mentoring the existing CATS in PST will reduce VF and prevalence and severity of CMDs at 48 weeks among ALHIV in Zimbabwe, compared with standard CATS care. The primary hypothesis is that the CATS-PST intervention will be more effective than standard CATS care in reducing the proportion of ALHIV who have died or who have VF at 48 weeks (defined as a viral load ⩾1000 copies/ml). The secondary hypotheses are that adolescents receiving the CATS-PST intervention will have (i) reduced prevalence and severity of depression and/or anxiety symptoms (using the SSQ-14 and PHQ-9 scales) and (ii) improved health-related quality of life, compared with adolescents receiving standard CATS care at 48 weeks after enrolment.

### Trial outcomes

#### Primary outcome

The proportion of adolescents with VF (defined as viral load >100 copies/ml) or death at 48 weeks (endline).

#### Secondary outcomes

- The proportion of adolescents with CMD symptoms (defined as SSQ-14 score ≥8), and the mean SSQ-14 and PHQ-9 scores.
- The proportion of adolescents with depressive symptoms (defined as PHQ-9 score ≥11).
- The mean total score for health-related quality of life measured using the EQ-5D.

In the original trial protocol, the cut-point for the CMD symptoms outcome was an SSQ-14 score ≥7, but a cut-point of ≥8 will be used here, as this was the optimal cut-off in a study involving adolescents in Zimbabwe with affective disorders (Langhaug *et al*., 2010).

### Trial setting

The trial will be conducted in 10 districts of Zimbabwe across five provinces: Mashonaland East (Murewa district), Midlands (Gokwe South and Kwekwe districts), Matabeleland North (Hwange district), Matabeleland South (Beitbridge, Gwanda and Matobo districts) and Masvingo (Chiredzi, Chivi and Zaka districts). In each district, the Zvandiri programme is operational in six health centres, four rural and two urban. The 60 health centres (all referred to as ‘clinics’ here) comprise 20 hospitals, 20 clinics and 20 rural health centres.

### Trial design

**Fig. 1.**
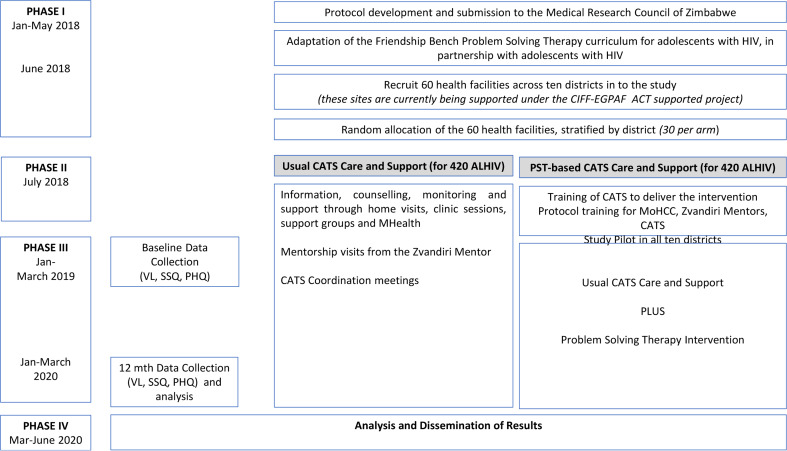
Study design.

The study will be a cluster-randomised controlled trial with health centre as the unit of randomisation. The 60 clinics will be randomised (1:1 allocation within the district) to either CATS-PST support or standard CATS support, stratified by 10. Randomisation will be performed by an independent statistician using a pre-written Stata do-file. Fourteen ALHIV aged 10–19 years will be recruited per clinic (total = 840) as described in the Baseline Procedures section (Fig. 1).

#### Eligibility criteria

- *Inclusion criteria*: ALHIV aged 10–19 years, eligible for ART (i.e. either starting or already on ART), with SSQ-14 score ≥7, and able to provide written informed assent for those aged 10–17 and their caregiver be able to provide written informed parental/caregiver consent. Those aged 18–19 years will be asked for written informed consent.
- *Exclusion criteria*: Participants will not be eligible if they are unable to comprehend the nature of the study in either English, Shona or Ndebele, are currently in psychiatric care, or end-stage AIDS, current psychosis, intoxication and/or dementia. All those excluded for medical reasons will be referred for appropriate care to one of two tertiary facilities in Harare.

### Screening and enrolment

From the 60 clusters, a list of potentially eligible participants will be created from the ALHIV already registered with Zvandiri. Mobilisation of potential participants will be done by CATS during home visits to encourage both the participant and caregivers (for minors aged 10–17 years) to come to the facility on set days. Additionally, potential participants who are not already registered with Zvandiri will be sensitised about the study in the Opportunistic Infection (OI) department. After pre-screening for eligibility, all potential participants and their caregivers will be invited to a trial orientation meeting where the details of the trial will be explained by an experienced Zvandiri mentor. Subsequently, potential participants will be screened for CMD symptoms by the research team using the SSQ-14. Eligible adolescents and caregivers who opt to take part in the trial will be asked to provide written consent and assent and will be booked for enrolment procedures and baseline assessments. Those who are not eligible for the trial will not be re-screened. Participants will be enrolled in the study over January to March 2019, in both arms concurrently

All participants attending clinics allocated to the control arm will receive Zvandiri standard care which entails ART, adherence support and counselling from clinic-based nurses and primary counsellors as set out in the prevailing Ministry of Health and Child Care (MoHCC) guidelines (Ministry of Health and Child Care, 2016), plus counselling and home-based support from trained, mentored CATS, monthly support groups, weekly short message services (SMS) and weekly home visits.

All participants attending clinics in the intervention arm will receive the usual Zvandiri standard care plus PST. CATS will be trained and receive continued supervision in the delivery of the PST intervention in this arm. In both arms, ALHIV presenting with the signs of psychological distress will be referred by CATS to the clinic as per standard MoHCC and Zvandiri procedures for further assessment and management. This will include follow-up by a trained mental health nurse, where available. Psychological distress is defined as a report of visual/auditory hallucinations or suicidal ideation on the SSQ-14, or a score of 21 or higher on the PHQ-9.

#### Intervention

The CATS-PST 6-session intervention will be adapted from the four-phase Friendship Bench programme (Verhey *et al*., 2015; Chibanda *et al*., 2016b). In the first phase, *kuvhura pfungwa* (opening the mind), the CATS will help the participant to understand what is happening in her or his life and encourage her or him to share what is going on and how she or he feels about it. In the second phase (*kusimudzira*), the participant is helped to choose one problem to work on, they will then help the participant brainstorm for possible solutions. In the third phase, *kusimbisa*, the CATS with the participant focuses on a detailed solution selection and devising a SMART action plan. In the last phase of *kusimbisisa*, participants who have completed 4–6 PST sessions are invited to take part in the 6-week Circle Kubatana Tose (Holding Hands Together) support group where people facing similar life challenges who have all gone through the counselling can share their stories and spend time together in a safe and protected environment while learning a craft skill (Chibanda *et al*., 2016c). The intervention will also adopt inputs gathered during the formative work to address the specific needs of the ALHIV.

The adapted intervention will be fully described in a separate paper. Sixty CATS (two per intervention clinic) will be trained by the Friendship Bench team in the newly adapted CATS-PST intervention. We will use the training model previously used for scaling up the Friendship Bench to over 70 primary care facilities (Chibanda *et al*., 2016c). The training programme lasts for 3 weeks and makes use of role play, pre and post-tests, group sessions and one-on-one sessions for those who have difficulties. The curriculum will include the use of PST with ALHIV and how to make referrals to mental health services. The CATS are currently working in these sites and have already been selected based on their readiness and capacity to provide peer counselling.

Weekly nurse-led group supervision and monthly supervision from a mental health specialist will be provided. The CATS will complete a brief evaluation on their experiences of delivering the intervention.

#### Sample size, power and randomisation

We aim to recruit 840 ALHIV, on ART from 60 clinics in 10 districts. Based on baseline results of the previous trial (Mavhu *et al*., 2020), we have assumed that 43% of participants will have VF at 48 weeks (endline) in clinics offering standard CATS support arm, within-clinic cluster coefficient of variation of 0.25, and 15% loss to follow-up (i.e. 14 participants recruited per clinic, with 12 seen at endline). This sample size provides 85% power to detect a 30% prevalence of VF in the CATS-PST support arm after 48 weeks (Table 1). The sample size also provides 85% power to detect a difference in proportion with VF of 36% in the standard CATS arm *v*. 24% in the CATS-PST arm. The sample size provides 87% power to detect a difference in the proportion with CMD symptoms at 48 weeks of 16% in the standard CATS arm and 8% in the CATS-PST arm.

**Table 1.** Power of the study if 840 ALHIV are recruited in 60 clinics (14 per clinic; 12 seen at 12 months)

% with VF in intervention arm	% with VF in control arm	Power
30%	43%	85%
31%	43%	78%
32%	45%	83%
24%	36%	85%

#### Data collection

A finger prick blood sample will be collected from all eligible recruited study participants at baseline and endline and transported to Harare Hospital as a dried blood spot sample for an HIV viral load test at the National Microbiology Reference Laboratory. CMD symptoms will be measured by the SSQ-14 which has been used extensively in Zimbabwe as a screening tool with reliable sensitivity and specificity (Chibanda *et al*., 2016a). The SSQ-14 is a dichotomous 14-item questionnaire, originally developed and validated in Shona (Chibanda *et al*., 2016a) and used largely at primary health care settings in Zimbabwe. Based on a validation study among ALHIV in Zimbabwe, we will use a cut-point of ≥8 which had a sensitivity of 85% and a specificity of 80% against a gold standard of major or minor depression with significant dysfunction on the Mini-International Neuropsychiatric Interview for Children and Adolescents (MINI-KID) (unpublished data). Clinical diagnosis of depression symptoms will be measured by the Patient Health Questionnaire (PHQ-9). The PHQ-9 is a nine-item Likert scale used to measure depression symptoms (Kroenke *et al*., 2001) and has been validated in Shona (Chibanda *et al*., 2016a). The PHQ-9 will be used as a secondary outcome measure for depression symptoms at 12 months with a cut-point of ⩾11 (Kroenke *et al*., 2001). Health, disability and health-related quality of life will be measured using the EQ-5D. We will also collect data on

- demographic and social characteristics including the level of caregiver and household support, food insecurity, HIV status disclosure
- experience of stigma
- clinical information including ARV regimen prescribed and experience of treatment switching, adherence practices.

The study has a qualitative component to answer critical questions around the feasibility of this peer-led intervention, the experiences of CATS when delivering the intervention, necessary modifications to the PST model itself in order to be delivered effectively by young people to adolescents and the support required by CATS. Young adolescents may have difficulty examining and articulating their experiences as well as listing their different problems when asked about them directly. For these adolescents, it may be more effective to elicit their problems using drawings. At endline, we will conduct two focus group discussions, each of about 10 participants, on their experiences of being supported by the CATS using PST. The discussion will be recorded and analysed. Participants will be randomly selected and give consent to join in the focus group discussions.

### Case reviews

As part of the intervention, all the CATS providing PST in the intervention arm will be visited by the Zvandiri Mentor on a weekly or bi-weekly basis to review individual cases. This review will include a case discussion, review of the case file documentation and discussion around the challenges and success experienced by the CATS. Twenty CATS (10 male and 10 female), two per district, will be purposively selected from the 60 intervention arm CATS for audio recording of these case reviews throughout the 12 months. These recordings will be transcribed verbatim, translated and analysed to provide an in-depth understanding of CATS experience of delivering PST for their clients, implementing fidelity and the range of problems and solutions identified by their clients.

### Audio diaries

At the beginning of the study, 10 CATS will be purposively selected and invited to record an audio over a 2-week period at three different time points throughout the study to describe their experiences of delivering the intervention to their clients. The CATS’ audios will be recorded in the language of their choice, transcribed verbatim and translated then analysed (Table 2).

**Table 2.** Enrolment, intervention and assessment schedule

Time	Jan–Mar 2019	Jan–Dec 2019	Jan–Mar 2020
Enrolment			
Eligibility screen	X		
Informed consent	X		
Interventions			
Standard CATS		X	
CATS-PST		X	
Assessments			
Viral load	X		X
Health-related quality of life	X		X
SSQ-14 cut-point of ≥8	X		X
EQ 5D	X		X
PHQ-9 cut-point of ≥11	X		X

#### Data management and analysis

An ODK form will be developed and loaded onto Android phones for data collection. Ten data clerks will be trained to capture data using the ODK questionnaire. Data will be uploaded to the cloud and exported to Stata for analysis.

Quantitative analysis will be at the individual level. The primary comparison will be the proportion of participants with VF or death at 48 weeks between arms. Binary outcomes will be estimated as prevalence ratios using random-effects logistic regression, adjusting for clinic as a random effect, district as a fixed effect, and for baseline viral load and baseline score of PHQ-9 or SSQ-14 as appropriate. Variables associated with loss-to-follow-up will also be adjusted for. For continuous outcomes, analogous analyses will be conducted using mixed-effects linear regression.

The focus group discussions, audio diaries and PST sessions will be recorded and transcribed for analysis. All case reporting forms, reports and study-related records will be identified by coded number to maintain confidentiality. All records will be kept in locked file cabinets, all computer entries will be done with coded numbers only with limited computer access to study personnel. Clinical information will not be released to any party without written permission and all patient identifiers removed. All recordings and drawings will be kept in a lockable cabinet, and destroyed after use.

#### Ethical considerations

This protocol will be subject to review and approval by institutional review boards at all participating institutions, including the Medical Research Council of Zimbabwe. Referral for social protection services if abuse or exploitation is uncovered at any point during the study will be made to the relevant authorities and or professionals. Adolescents in the control group who present with depression will be managed by their existing clinic services. Participants referred for further care will not be excluded from the study but will receive the extra input from either a psychiatrist or clinical psychologist in addition to the PST, while in the control arm, individuals will be referred to a tertiary facility offering psychiatric services as part of standard care in the event that the clinic staff are unable to manage the cases. Once the study has been completed, it is expected that CATS in the control arm clinics will also be trained in the PST intervention, so that control arm participants also receive the same intervention, if it is found to be effective.

Africaid and the study team will always adhere to the best interest of the child principle. This study will involve highly vulnerable adolescents – those living with HIV and mental illness, and CATS who themselves are living with HIV and their right to confidentiality, privacy, respect and access to services will always be upheld. Written informed consent and assent will be obtained from all study participants prior to enrolment in the study. If participants are under 18, assent will be obtained along with written parental or caregiver consent. The written assent and consent forms will include assent and consent for any photographs and/or personal stories collected and shared throughout the duration of the study. Patient identifying information will not be collected. At the time of analysing data and publication of the results of the study, the name or identity of participants will not be used. Participants wishing to withdraw from the study will be allowed to do so with no effect on their subsequent care.

## Discussion

To our knowledge, there is one pilot trial (Betancourt *et al*., 2017) and one published protocol of a trial (Sam-Agudu *et al*., 2017) to improve mental health among ALHIV in Africa. A pilot randomised controlled trial in Rwanda found that children aged 7–17 who were affected by HIV benefitted from family-based mental health promotion. However, only 21 children (12% of the sample) were living with HIV. In Nigeria, a planned trial of coordinated transition from paediatric to adult HIV care includes mental health as a secondary outcome.

Our trial is the first to prospectively enrol ALHIV who screen positive for CMD symptoms. The results of the study will show whether it is feasible for CATS to deliver PST to their peers on ART who have symptoms of CMD. The difference in SSQ-14 and PHQ-9 score will show the effectiveness of this intervention on CMD symptoms among ALHIV in Zimbabwe.

This research aims to address key challenges related to ART adherence and virological suppression for ALHIV. The trial has several methodologic strengths. It is large, well powered and representative of the whole country. A cluster-randomised design was used because the intervention is implemented through training CATS who are already based at specific clinics. The trial has a biological endpoint (viral load) as well as the secondary outcomes of CMD symptoms and quality of life. As a limitation, complete blinding is impossible. Data analysts will be unaware of which clinics within a district share a trial arm, and which arm is which, until after the publication of an analytical plan.

Operationally, the greatest strength of the trial is the network of CATS already integrated into primary care clinics across Zimbabwe, with training and mentoring programme by Africaid. A cluster-randomised controlled trial of the effectiveness of CATS care compared to clinic-based care on virological suppression has recently been completed (Mavhu *et al*., 2020). A logistic limitation is that some adolescents will have mental health problems requiring more complex treatment than CATS can provide, including medication. A referral mechanism is in place, but mental health care availability is limited and inequitable.

The Friendship Bench programme was originally developed in Harare and delivered by health advisors who were primarily older women. A cluster-randomised controlled trial in adults found it to be highly effective at reducing symptoms of CMDs and depression (Chibanda *et al*., 2016d). Importantly, it was equally effective for the 40% of trial participants who were living with HIV, and for the youngest participants (aged 18–22) (Chibanda *et al*., 2016c). This supports the theory that an adaptation of the programme will benefit ALHIV. Lessons learned from the scale-up of Friendship Bench and its expansion into rural areas show that working with a dynamic group of younger people who have different life challenges than those of the lay health workers bring in a new dimension in training younger people to be peer counsellors.

## Consent for publication

There will be a statement of consent to be audio taped for adolescents in the study. Those aged 18 and 19 years will give adult consent and in case of younger participants (10–17 years) both the child and parental or caregivers consent will be sought.
